# Sustained wakefulness and visual attention: moderation by chronotype

**DOI:** 10.1007/s00221-016-4772-8

**Published:** 2016-09-13

**Authors:** Nicola L. Barclay, Andriy Myachykov

**Affiliations:** 1 Sleep and Circadian Neuroscience Institute (SCNi), Nuffield Department of Clinical Neurosciences, Sir William Dunn School of Pathology, University of Oxford, South Parks Road, Oxford, OX1 3RE UK; 2 Department of Psychology, Faculty of Health and Life Sciences, Northumbria University, Newcastle, UK; 3 Center for Cognition and Decision Making, Higher School of Economics, Moscow, Russia

**Keywords:** Attention, Chronotype, Sleep deprivation, Visual attention, Wakefulness

## Abstract

**Introduction:**

Attentional networks are sensitive to sleep deprivation and increased time awake. However, existing evidence is inconsistent and may be accounted for by differences in chronotype or time-of-day. We examined the effects of sustained wakefulness over a normal “socially constrained” day (following 18 h of sustained wakefulness), following a night of normal sleep, on visual attention as a function of chronotype.

**Methods:**

Twenty-six good sleepers (mean age 25.58; SD 4.26; 54 % male) completed the Attention Network Test (ANT) at two time points (baseline at 8 am; following 18-h sustained wakefulness at 2 am). The ANT provided mean reaction times (RTs), error rates, and the efficiency of three attentional networks—alerting, orienting, and executive control/conflict. The Morningness–Eveningness Questionnaire measured chronotype.

**Results:**

Mean RTs were longer at time 2 compared to time 1 for those with increasing eveningness; the opposite was true for morningness. However, those with increasing morningness exhibited longer RT and made more errors, on incongruent trials at time 2 relative to those with increasing eveningness. There were no significant main effects of time or chronotype (or interactions) on attentional network scores.

**Conclusion:**

Sustained wakefulness produced differential effects on visual attention as a function of chronotype. Whilst overall our results point to an asynchrony effect, this effect was moderated by flanker type. Participants with increasing eveningness outperformed those with increasing morningness on incongruent trials at time 2. The preservation of executive control in evening-types following sustained wakefulness is likely driven by differences in circadian phase between chronotypes across the day.

## Introduction

Sufficient sleep is necessary to maintain high levels of cognitive functioning during the waking period. Sleeping consistently less than 7 h has been associated with cumulative impairments in vigilant attention and alertness (Belenky et al. 2003), and experimental studies consistently show general impairments in vigilant attention following sleep restriction or sleep deprivation (Basner and Dinges 2011; Basner et al. 2011; Lim and Dinges 2008). It has been proposed that decrements in vigilant attention following sleep deprivation lay the foundation for impairments in other cognitive domains (Lim and Dinges 2008). At the same time, visual attention is a complex system of functionally and anatomically distinct brain networks, which underlie our ability to (1) maintain an “alert” state (alerting network), (2) “orient” attention to stimuli (orienting network), and (3) resolve conflict when numerous stimuli simultaneously compete for attention (executive control network) (Fernandez-Duque and Posner 2001; Petersen and Posner 2012). These networks are subserved by distinct neurobiological pathways (Fan and Posner 2004), which are likely differentially affected by sleep and extended periods of sustained wakefulness such as following sleep deprivation. Existing studies demonstrate *general* impairments in vigilant attention (akin to “alerting”) following sleep restriction or deprivation (e.g. Lim and Dinges 2008). However, few attempt to disentangle the effect of sleep deprivation or sustained wakefulness on individual attentional networks, and those that have report inconsistent findings (e.g. Jugovac and Cavallero 2012; Martella et al. 2011; Muto et al. 2012; Roca et al. 2012; Trujillo et al. 2009). In part, these inconsistencies may stem from uncontrolled circadian-variability, i.e. differences in the phase position of the circadian rhythm (an endogenous “biological clock” which dictates the timing of our physiological and behavioural processes) due to either inter-individual differences in chronotype, or time-of-day effects.

Chronotype refers to the tendency towards morningness or eveningness in the preferred timing of daily activities and sleep (Levandovski et al. 2013). Morning-types, the so-called larks, find it easy to arise in the morning, function best at this time, and fall asleep easily during early evening. Evening-types, on the other hand, the so-called owls, find it hard to get up early, are at their peak during late evening, and go to bed late, often in the early hours of the morning. Morning-types and evening-types have been shown to differ in the endogenous phase of the circadian rhythm (Baehr et al. 2000; Kerkhof and Van Dongen 1996); their homoeostatic regulation of sleep (Mongrain et al. 2005, 2006, 2011); and response to sleep fragmentation and deprivation (Mongrain et al. 2008; Taillard et al. 2011). These inter-individual differences between chronotypes influence behavioural differences in sleep timing and the profile of neurobehavioural functioning.

Furthermore, circadian-variability differentially affects neurobehavioural functioning, particularly attention, at different times of day (Valdez et al. 2012). This effect is likely due to circadian mechanisms driving alertness. Generally speaking, behavioural alertness and vigilant attention are subject to dynamic circadian variation across the normal waking day independent of increasing sleep pressure (our homoeostatic response to accumulated time awake, driving sleepiness). Neurobehavioural functioning is poor in the morning following awakening, which steadily improves across the day with an early evening peak (~6–9 pm), during which alertness and performance are relatively stable, which then progressively declines into the night (Goel et al. 2011; Mollicone et al. 2010). The circadian rhythm of neurobehavioural functioning largely parallels that of the core body temperature rhythm (Monk et al. 1997). However, the timing of these peaks and troughs in neurobehavioural functioning throughout the normal waking day is likely to vary as a function of chronotype, given the inter-individual differences in the timing of circadian phase. Indeed, Matchock and Mordkoff (2009) demonstrated differential effects of both chronotype and time-of-day on the efficiency of the attentional networks. Whilst the efficiency of the alerting network differed between morning/neither types and evening-types in the latter half of the day, the orienting network showed no chronotype or time-of-day effects; and the executive control network was consistent across chronotypes, but demonstrated peaks at midday and mid-afternoon. However, the grouping of morning/neither types compared to evening-types assumes similar phase timing between morning and neither types, rather than considering the possibility that differences between chronotypes may vary incrementally across the chronotype spectrum. Thus, to gain a more complete understanding of the influence of sustained wakefulness on attention, one must consider (1) attention as a multifaceted construct; (2) the influence of chronotype across the whole spectrum; and (3) the time-of-day of testing. That said, Matchock and Mordkoff (2009) examined differences in attention across only a 12-h day (testing at 08:00, 12:00, 16:00 and 20:00 h). If we are to assume that non-shift-workers who sleep in one primary nocturnal sleep bout typically remain awake around 16–18 h (given that the average sleep duration of adults is around 6–8 h; see Bin et al. 2012, for a review), it remains to be determined whether attentional efficiency diverges by chronotype in the later evening beyond 20:00 h (compared to morning testing). Indeed, others have demonstrated that cumulative effects of sleep restriction and deprivation on behavioural alertness begin to emerge after 16 h of sustained wakefulness (Van Dongen et al. 2003). Nevertheless, it remains unclear whether this pattern of response to sustained wakefulness is consistent across the full chronotype spectrum. Hypothetically, individuals with a tendency towards morningness might show enhanced attentional efficiency in the morning relative to both those with a tendency towards eveningness and evening testing. Attentional efficiency, on the other hand, could be preserved in those with eveningness tendencies during the late evening relative to both those with morningness tendencies and morning testing. These expectations follow a “synchrony effect” whereby evening-types perform better on a range of cognitive tasks in the evening and morning-types in the morning (see Adan et al. 2012, for a review; Horne and Östberg 1980; Kerkhof 1985; May and Hasher 1998; May et al. 1993). The effect may occur because the timing of the task coincides with peak levels of alertness, higher core body temperature, and arousal. Indeed, under socially constrained conditions (such as when one has to awaken early in the morning to commute to work), evening-types awaken closer to their body temperature nadir, when alertness is low (Baehr et al. 2000; Waterhouse et al. 2001), thus contributing to impaired performance relative to morning-types. On the other hand, during the evening, the onset of melatonin occurs at an earlier clock time in morning-types (Lack et al. 2009), decreasing alertness and contributing to poorer neurobehavioural performance compared to evening-types.

These considerations suggest that an investigation of the potential interactive impact of circadian-variability (chronotype and time-of-day) on the attentional networks’ functioning during sustained wakefulness is not complete without considering the corresponding effects across the total time frame of a typical waking day (i.e. 18 h of sustained wakefulness). Furthermore, whilst sleep restriction and sleep deprivation studies (utilising the constant routine and forced desynchrony protocols) have elegantly demonstrated the opposing forces of the circadian rhythm and homoeostatic sleep drive on neurobehavioural functioning (see Goel et al. 2013, for a review), the ecological validity of such studies is debatable. It is important to consider the potential interactive impact of circadian-variability on attentional functioning on a real-world level. The study reported below examines the influence of chronotype and time-of-day on the functional efficiency of the attentional networks under a typical “socially constrained” day (i.e. 8 am–2 am the following morning; 18 h of sustained wakefulness) following a night of normal sleep in good sleepers.

## Methods

### Participants

Participants were recruited from the general population of the north-east of England through poster advertisements, emails to staff and students of Northumbria University, and through social media. Thirty participants initially volunteered for the study; 26 provided complete data. All participants were self-reported good sleepers (mean Pittsburgh Sleep Quality Index [PSQI], Buysse et al. 1989, =3.54 [1.61]); were aged between 18 and 40 years of age (mean age 25.58; SD 4.26; 54 % male); did not have a history of/or current sleep, medical or psychiatric disorder, or drug/alcohol abuse; were not taking medications (including recreational drugs) which could affect their sleep; were not experiencing excessive daytime sleepiness (mean Epworth Sleepiness Scale [ESS], Johns 1992, score = 3.96 [2.46]); were not shift-workers; had not travelled across three time zones in the previous 3 months; had normal (or corrected-to-normal) vision; and were non-smokers. Participants were rewarded with a £40 “Love2Shop” voucher for their participation.

### Measures

#### Chronotype

The Horne and Östberg Morningness–Eveningness Questionnaire (MEQ: Horne and Östberg 1976) was used to assess chronotype. The MEQ includes 19 self-reported items assessing preferred timing of daytime activities, sleep habits, hours of peak performance, and times of “feeling best” and maximum alertness. Responses are combined to provide a total score ranging between 16 and 86 with higher scores indicating a greater tendency towards morningness. We examined the continuous morningness–eveningness scale rather than extreme groups of morning- versus evening-types for greater power and to best represent the full chronotype spectrum.

#### Attention

**Fig. 1:**
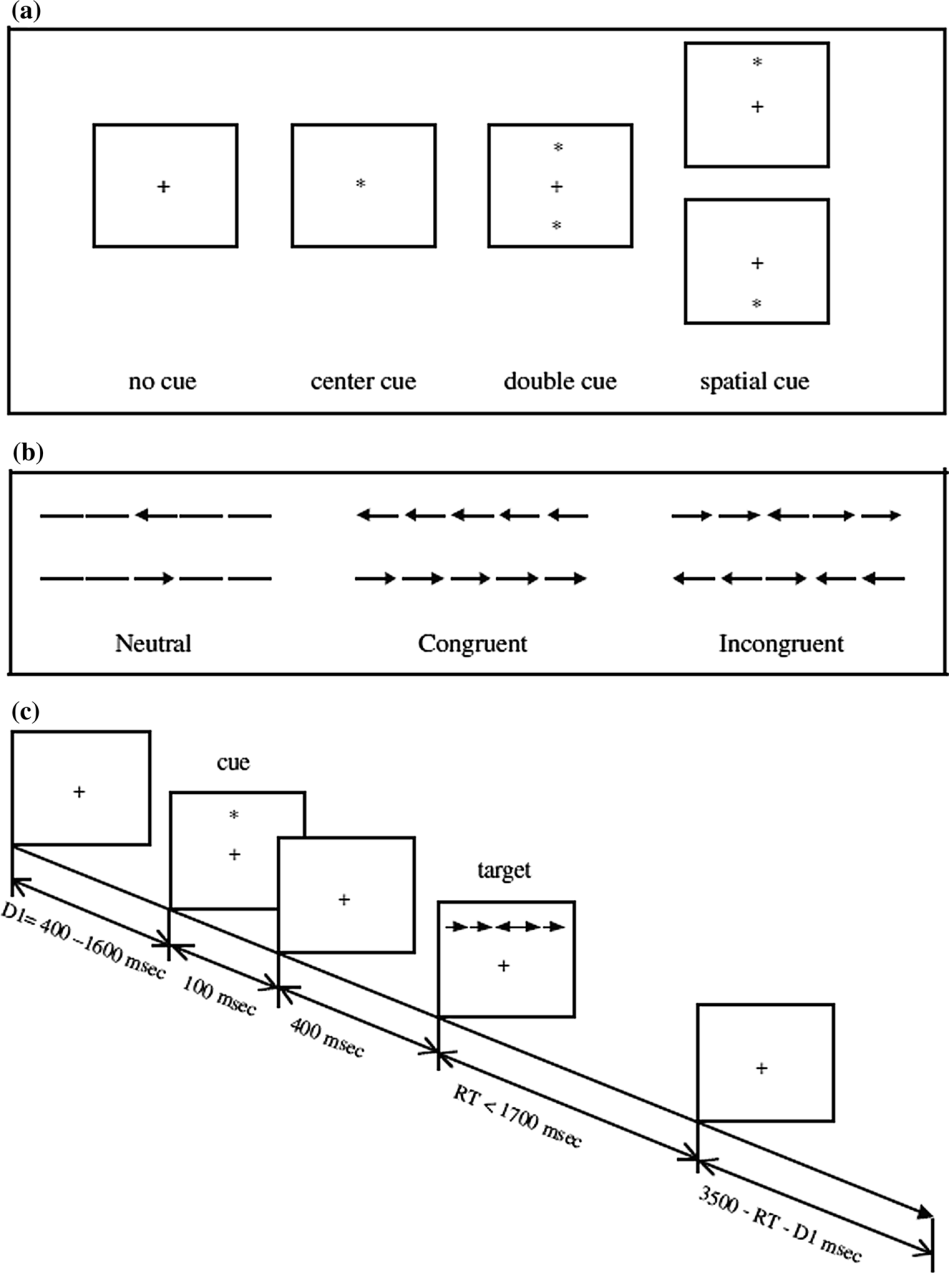
ANT procedure. **a** The four cue conditions, **b** the flanker types, and **c** an example of the procedure. Reprinted with permission from Fan et al. (2002)

The Attention Network Test (ANT: Fan et al. 2002) was used to examine the attentional networks’ performance (see Fig. 1). In the ANT, participants perform on centre-, double-, spatial-, or no-cue trials (100 ms) between two central fixation events. At the second central fixation (400 ms), the target arrow (left or right) is presented either above or below the fixation cross, and it is either presented alone (*neutral* condition), with two flankers either side pointing in the same direction (*congruent* condition), or with two flankers either side pointing in the opposite direction (*incongruent* condition) (lasting no longer than 1700 ms). Upon presentation of the target, participants are required to indicate by pressing designated keys on a computer keyboard whether the corresponding arrows point leftwards or rightwards. The ANT provides a raw reaction time (RT) measure for each of the conditions (cue type: no cue, centre cue, double cue, spatial cue; flanker type: neutral, congruent, incongruent). Additionally, the ANT provides specific measures of alerting, orienting and conflict resolution (executive control). The *alerting* score is calculated by subtracting the mean RT of the double-cue conditions (which alerts the participant to the imminent target, but does not provide information on its location either above or below the cross) from the mean RT of the no-cue conditions. The *orienting* score is calculated by subtracting the mean RT of the spatial-cue conditions (which alerts participants to the imminent target and provides information on its location) from the mean RT of the centre-cue conditions (which only alerts participants to the imminent target at one location). The *conflict* (executive control) score is calculated by subtracting the mean RT of all congruent flanked conditions from all incongruent flanked conditions (from all cue types). Greater scores typically indicate increase in processing difficulty: (a) maintaining alertness without a cue (alerting); (b) disengaging from the centre cue (orienting); or (c) resolving conflict (executive control) (Fan and Posner 2004). We first examined overall reaction times from the ANT as a function of cue type and flanker type, as well as chronotype and time-of-day; followed similarly by examination of error rates; and finally of attention network scores as a function of chronotype and time-of-day.

### Procedure

The procedure for the present study comprised 3 stages.

#### *Stage 1: Participant pre*-*screening*

Prospective participants completed a brief screening questionnaire sent via email to confirm eligibility to participate. The screening questionnaire contained questions on general health, demographics, the Pittsburgh Sleep Quality Index (Buysse et al. 1989), the Epworth Sleepiness Scale (Johns 1992), and the Morningness–Eveningness Questionnaire (Horne and Östberg 1976). Eligible participants (good sleepers, no excessive sleepiness) completed an informed consent form and continued onto stage 2 of the study.

#### Stage 2: Week prior to sustained wakefulness protocol (SWP)

One week prior to the SWP, participants met with the researcher at the Northumbria Centre for Sleep Research, Northumbria University, to be informed of the risks and requirements of the protocol, to be provided with a questionnaire booklet outlining the protocol, and to familiarise themselves with the ANT task. Throughout the protocol, participants were informed that during and 6 h following the SWP, they must not drive, use tools/operate machinery, or perform any tasks that are demanding or may jeopardise their safety (i.e. running a bath), and that they must not do any of these until they had slept at least 6 h. Participants were provided with a one-week sleep diary to record their sleep patterns and an actiwatch (a watch-like device that records data on movement, and hence information on sleep–wake activity) to be worn during the week prior to the SWP to ensure that all participants did not suffer from a circadian rhythm disorder, or were excessively sleep-deprived prior to and during the SWP. Participants were required to continue to wear the actiwatch during the SWP to ensure adherence. During the week prior to the SWP, participants were required to adhere to their normal routine as much as possible; e.g. have a normal sleep duration (approximately 7.5–8.5 h per day) and maintain regular sleep schedule (e.g. approximate bed time of 11:30 pm ± 60 min, and waking up at approximately 7:30 am ± 60 min each night/morning). Only data from participants who obtained sufficient sleep (>6 h) and adhered to a stable sleep schedule the week prior to the SWP were included in the analyses. All recruited participants adhered to these requirements (actigraphy data unreported; available upon request from the first author).

#### Stage 3: Sustained Wakefulness Protocol (SWP)

- Day 1, Morning session (7:45 am) (performed in participants’ homes): Participants were required to awaken at 7:30 am and were called by the researcher by telephone at 7:45 am to confirm that they were ready to commence the SWP and to be given instructions. Participants continued to wear the actiwatch. Participants were instructed that they should not have consumed breakfast, caffeine, alcohol or used recreational drugs prior to this session (or alcohol/recreational drugs the night before) and should not have performed any intense physical exercise. Participants were instructed to sit at their computer (connected to the Internet) to commence the ANT at 8 am. Upon completion, participants were free to go about their day as normal. Participants were required to refrain from consuming caffeine, alcohol or using recreational drugs during the protocol, should not perform any intense physical exercise, and should not take any naps (compliance confirmed by actigraphy).
- Day 1, Evening session (7:45 pm): Twelve hours following the initial wake up time participants were called by the researcher to confirm that they had adhered to the protocol so far, that they were prepared to remain awake for the following 6-h period at home, and that there was someone home with them to act as their emergency contact in case their safety was compromised. Every hour the researcher called participants to ensure compliance to the protocol and to ensure their safety to continue.
- Day 2, Early morning session (2 am): Eighteen hours following the beginning of the SWP, participants were called by the researcher to confirm that they had adhered to the protocol so far and that they were prepared to remain awake and to be given instructions. Participants were instructed to sit at their computer (connected to the Internet) to commence the ANT. Upon completion, participants called the researcher to monitor their health and safety. Participants were then permitted to retire to bed.
- Day 2, Later morning: Upon awakening the next morning, participants called the researcher to ensure their safety to go about their day. Participants were then debriefed about the nature and aims of the study by phone, and a debrief sheet was also sent by email.

### Compliance with ethical standards

The present study received full ethical approval from the Northumbria University Faculty of Health and Life Sciences Ethics Committee and has therefore been performed in accordance with the ethical standards laid down in the 1964 Declaration of Helsinki. Informed consent was obtained from all individual participants included in the study.

## Results

Mean chronotype score was 51.54 (SD 6.99; range 36–63). Mean ANT reaction times were pooled from all correct trials for all participants (*n* = 26). Incorrect trials accounted for 8.69 % of the total trials. Additionally, trials with RT ± 2SD from the overall mean RT were excluded from analyses (6.25 %). Table 1 shows the mean RT and SD, and Table 2—the mean error rates and SD, for each of the experimental conditions (4 × cue type; 3 × flanker type) for time 1 (before sustained wakefulness; 8 am) and time 2 (following 18-h sustained wakefulness; 2 am). The distribution of RT scores across participants deviated from normal for 15 out of 24 conditions (Kolmogorov–Smirnov test <.05); thus, reciprocal transformations were applied to all variables prior to analysis (resulting in 3/24 variables deviating from normality). For those that still deviated from normality following transformation, skew and kurtosis were significantly reduced (skew range −.72 to −.90 [SE = .46]; kurtosis range −.49 to .96 [SE = .89]).

**Table 1:** Mean RT (SD) for each experimental condition of the ANT by time (correct trials only and outliers ± 2SD excluded)

Flanker types	Time	Cue			
		No cue	Centre	Double	Spatial
Neutral	1	569.80 (87.78)	522.35 (98.38)	521.98 (91.27)	504.52 (98.83)
	2	571.86 (94.57)	527.55 (104.80)	519.64 (105.38)	501.73 (106.90)
Congruent	1	604.62 (94.37)	573.99 (85.55)	569.39 (95.94)	539.85 (95.02)
	2	616.01 (97.89)	585.94 (112.24)	571.80 (104.83)	547.82 (97.07)
Incongruent	1	710.30 (96.83)	701.95 (108.75)	687.66 (84.19)	645.25 (104.79)
	2	713.18 (88.24)	711.48 (108.41)	698.47 (98.07)	649.09 (108.88)

*ANT* Attention Network Test; Time 1 = before sustained wakefulness (8 am); Time 2 = following 18-h sustained wakefulness (2 am)

**Table 2:** Percentage of errors (SD) for each experimental condition of the ANT by time

Flanker type	Time	Cue			
		No cue	Centre	Double	Spatial
Neutral	1	6.30 % (24.32 %)	6.58 % (24.80 %)	5.64 % (23.08 %)	6.82 % (25.22 %)
	2	7.41 % (26.21 %)	7.53 % (26.40 %)	7.23 % (25.92 %)	7.07 % (25.65 %)
Congruent	1	5.62 % (23.04 %)	6.12 % (23.99 %)	5.59 % (23 %)	6.16 % (24.05 %)
	2	6.98 % (25.50 %)	7.39 % (26.17 %)	6.65 % (24.93 %)	7.45 % (26.28 %)
Incongruent	1	11.34 % (31.73 %)	13.22 % (33.89 %)	10.83 % (31.10 %)	9.69 % (29.60 %)
	2	14.76 % (35.49 %)	15.31 % (36.03 %)	15.21 % (35.94 %)	12.13 % (32.67 %)

*ANT* Attention Network Test; Time 1 = before sustained wakefulness (8 am); Time 2 = following 18-h sustained wakefulness (2 am)

### Mean reaction time

A repeated-measures ANCOVA was performed on mean (transformed) RT (reaction time), with time (1 = before sustained wake; 2 = following sustained wake), cue (no cue, centre, double, and spatial) and flanker type (neutral, congruent, and incongruent) as within-subject factors, and chronotype as covariate. Assumptions of sphericity were not met for the main effects of cue, flanker type, or the cue × flanker type interaction. In these cases, Greenhouse–Geisser correction to degrees of freedom was employed. There were significant main effects of cue (*F*[1.90, 72] = 5.28, *p* < .01, *η* ^2^ = .18) and time (*F*[1, 24] = 10.27, *p* < .01, *η* ^2^ = .30) on mean RT. Longer reaction times were exhibited on trials with no cue vs. all other cues and at time 2 versus time 1.

**Fig. 2:**
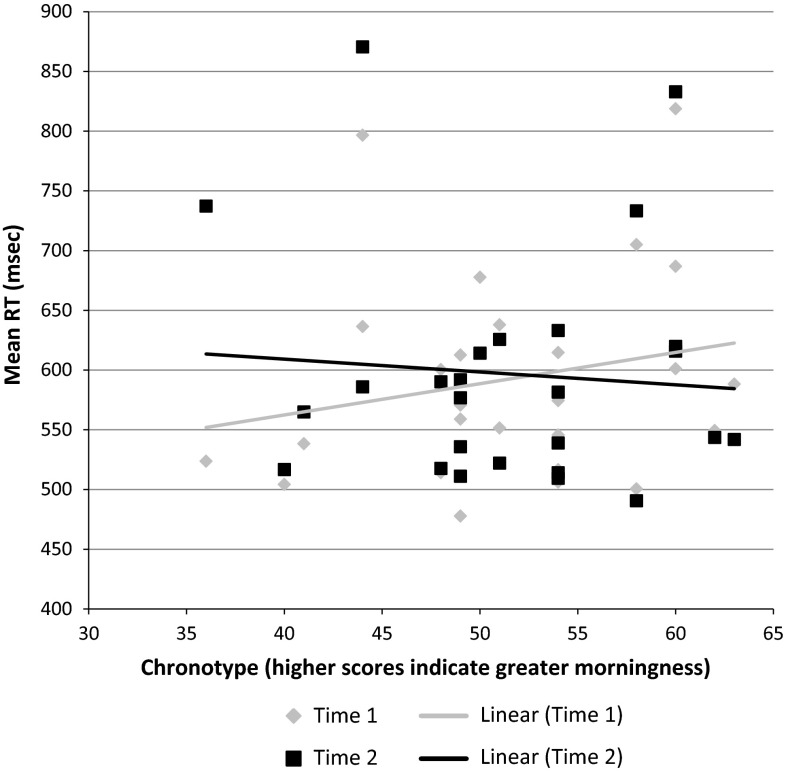
Scatterplot of overall mean reaction times (ms) at time 1 and time 2 by chronotype

The time × chronotype interaction was significant (*F*[1, 24] = 10.16, *p* < .01, *η* ^2^ = .30) as were the flanker type × time (*F*[2, 48] = 3.43, *p* < .05, *η* ^2^ = .13) and flanker type × time × chronotype interactions (*F*[2, 48] = 3.64, *p* < .05, *η* ^2^ = .13). Figure 2 displays the time × chronotype interaction. Mean reaction times were longer at time 2 compared to time 1 for those with increasing tendency towards eveningness, whereas the opposite was true for morningness where reaction times were longer at time 1. For the flanker type × time interaction, mean reaction times were longer on both congruent and incongruent (vs. neutral) trials at time 2 compared to time 1.

**Fig. 3:**
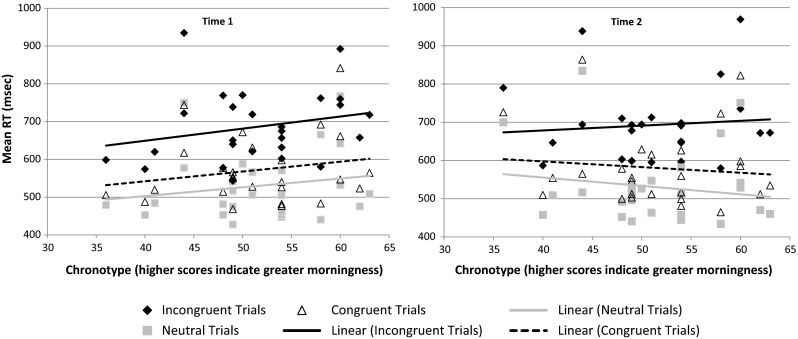
Scatterplots of mean reaction times (ms) split by congruency type, at time 1 and time 2 by chronotype

Figure 3 displays the flanker type × time × chronotype interaction. Whilst at time 1 reaction times across all flanker type trial types were longer with increasing morningness (relative to eveningness), at time 2 reaction times were longer on incongruent trials only in those with increasing morningness (relative to eveningness). In other words, evening-types outperformed morning-types on incongruent trials at time 2.

### Error rates

Table 2 shows the mean percentage of error rates and SD for each of the experimental conditions for time 1 (before sustained wakefulness; 8 am) and time 2 (following 18-h sustained wakefulness; 2 am). In order to examine error rates, we used generalised estimating equations (GEE, e.g. Hardin and Hilbe 2003). Unlike ANOVA, GEE allows for specifying distribution and link functions that are appropriate for analysing categorical frequencies. Here, we used a binomial distribution and logit link function (cf. Jaeger 2008) to model proportions of correct trials as a function of cue type (no cue, centre cue, double cue, and spatial cue) and flanker type (neutral, congruent, and incongruent) as within-subject variables, and chronotype as a covariate.

All main effects were non-significant; however, there were significant interactions: time × cue × flanker type (_GS_ *Χ* ^2^ = 5.39(1), *p* = .02) such that significantly more errors were made at time 2 for centre cues on incongruent trials only; cue × flanker type × chronotype (_GS_ *Χ* ^2^ = 4.39(1), *p* = .04) such that more errors were made on the centre-cue incongruent trials with increasing tendency towards morningness; and time × cue × flanker type × chronotype (_GS_ *Χ* ^2^ = 5.57(1), *p* = .02) such that more errors were made at time 2 for the centre cue on incongruent trials with increasing tendency towards morningness.

### Attentional network scores

The three attentional network scores (see Table 3) were calculated from correct trials (and after excluding +−2SD) as follows: alerting = (mean RT no-cue trials) − (mean RT double-cue trials); orienting = (mean RT centre-cue trials) − (mean RT of spatial-cue trials); conflict = (mean RT incongruent trials) − (mean RT congruent trials). Paired samples *t* tests revealed no significant differences in attentional network scores from time 1 to time 2 (all *p*’s > .05). A series of repeated-measures ANCOVAs were additionally run to examine the influence of chronotype as a covariate on attentional network scores from time 1 to time 2. No main effects or interactions were significant, though it is worth noting a trend towards significance on the time × chronotype interactions on both orienting and conflict scores (*p* = .08 and .07, respectively). Evening-types exhibited lower orienting scores at time 2 compared to time 1, whereas the opposite was true for morning-types. For conflict, evening-types exhibited lower scores at time 2 compared to time 1, whereas scores were equivalent at both time points for morning-types.

**Table 3:** Mean (SD) attentional network scores from time 1 to time 2

	Time 1	Time 2
Alerting	6.42 (22.60)	11.69 (19.03)
Orienting	65.03 (30.09)	67.47 (24.68)
Conflict	114.33 (41.45)	112.66 (31.08)

Time 1 = before sustained wakefulness (8 am); Time 2 = following sustained wakefulness (2 am)

## Discussion

Primarily, the present results demonstrate that 18 h of sustained wakefulness produced differential effects on visual attention as a function of chronotype, at least in terms of reaction time on a probe detection ANT task. Individuals with a tendency towards eveningness exhibited slower reaction times following 18 h of sustained wakefulness compared to initial testing at 8 am, whereas individuals with a tendency towards morningness exhibited the opposite. This finding is contrary to expectation based on the wealth of literature showing a “synchrony effect” (see Adan et al. 2012, for a review; Horne and Östberg 1980; May and Hasher 1998; May et al. 1993). Such an “asynchrony effect” (where performance is better at non-optimal time-of-day) may relate to the concept of “social jetlag”. Social jetlag refers to the misalignment between the endogenous circadian rhythm and time constraints imposed by social commitments (Wittmann et al. 2006). Forced awakenings at times out-of-sync with the biological drive for wakefulness impose physiological sensations akin to jetlag. Forcing evening-types to arise at 7:30 am in readiness for an 8 am test imposes an increased sleep debt on such individuals compared to morning-types, the latter of whom may have obtained a greater amount of sleep the night prior. Thus, at the subsequent 2 am testing, evening-types may have incurred an increased sleep debt which could account for the unexpected finding of longer RT at time 2 compared to time 1 for evening-types (though it is worth noting that all participants obtained at least 6 h sleep prior to testing).

An alternative explanation for the observed “asynchrony effect” is that sustained wakefulness on attention is modulated by compensatory mechanisms such as motivation or effort. At times of decreased efficiency and alertness, morning-types may need to employ greater effort allowing them to perform well on attentional tasks despite their decreasing arousal. Such a mechanism has been suggested to account for the absence of synchrony effects in relation to tasks that require a wide range of cognitive resources (Natale et al. 2003). However, to the best of our knowledge, this is the first demonstration of an asynchrony effect in attentional performance following sustained wakefulness. In a study of synchrony effects on general intelligence and visual-spatial and linguistic abilities, Freudenthaler and Neubauer (1992) demonstrated that level of motivation modulated the effects of chronotype and time-of-day on performance. The present data further suggest that a motivational mechanism may account for the asynchrony effect present for even bottom-up attentional processes. Others have found evidence of an asynchrony effect for tasks involving insightful problem solving, spatial aptitude, and implicit learning (Delpouve et al. 2014; Song and Stough 2000; Wieth and Zacks 2011). Interestingly, tasks related to the production of a well-learned pre-potent response rather than its inhibition demonstrate no synchrony/asynchrony effects (May and Hasher 1998). Differences between studies in evidence of synchrony/asynchrony effects are likely due to differences in the complexity of the tasks examined, or in the cognitive domain they employ.

Our data further suggest that the synchrony/asynchrony effect may depend on flanker congruency—on incongruent trials, those with increasing eveningness outperformed those with increasing morningness at time 2—evidence of a synchrony effect. Further, on examination of the error rate data, those with increasing morningness made more errors on centre-cue incongruent trials at time 2 than those with increasing eveningness. Our ANT data also indicate that evening-types exhibited a trend towards lower conflict scores (indicative of enhanced ability to resolve conflicting information) at time 2 compared to time 1, whereas the opposite was true of morning-types. Together, these results suggest that following 18 h of sustained wakefulness evening-types may be better at tasks of conflict resolution (which require inhibitory attentional processes) than morning-types. Strongest time-of-day effects are often observed for executive control of inhibitory functions compared to simple response tasks (Lustig et al. 2007), and synchrony effects are particularly evident on higher-order cognitive tasks requiring controlled processing of distracting stimuli (May 1999; May and Hasher 1998). In such studies, participants are worse at inhibiting distracting stimuli at their non-optimal time-of-day than at their time of optimal functioning.

It is likely that differential performance on inhibitory processing between chronotypes is due to differences in relative brain activation across the day. Schmidt and colleagues demonstrated that responses of brain regions known to be involved in monitoring and resolving cognitive conflict and error processing, in particular the anterior cingulate cortex and insula (Botvinick et al. 2001; Fan et al. 2003; Roberts and Hall 2008), are elevated in evening-types, but decreased in morning-types during the evening (Schmidt et al. 2012). Decrements in inhibitory control across the day may also be related to changes in frontal functioning (May and Hasher 1998). The lateral prefrontal cortex is, amongst other sites, involved in executive control of attention and modulated by dopamine (Fan and Posner 2004). Frontal areas are most vulnerable to the effects of sleep deprivation, demonstrating overall reduced activation as well as decreased response to cognitive tasks (Cajochen et al. 1999; Habeck et al. 2004; Jones and Harrison 2001). Interestingly, the effect of sleep deprivation on frontal areas, and particularly executive control functions, reflects individual differences and appears to be modulated by genes implicated in chronotype (Groeger et al. 2008; Vandewalle et al. 2009). Individuals with a genetic polymorphism in the PER3 clock gene predictive of morningness (5/5 tandem-repeat) are more vulnerable to executive control deficiencies following sleep loss (Groeger et al. 2008). The present results are congruent with these findings suggesting that those with a preference towards morningness show greater impairments in executive control following sustained wakefulness and that this difference (compared to eveningness tendencies) is likely due to functional deficiencies in frontal activation, driven at the molecular level by underlying genetic variability between chronotypes.

It is not possible, however, to dissociate whether this preservation in executive control in those with a tendency towards eveningness (relative to morningness) at time 2 is due to differential build-up of homoeostatic sleep pressure (i.e. increasing sleep propensity) over the waking period or differences in circadian phase between chronotypes. One could hypothesise that, given that frontal cortical areas are thought to be most sensitive to variations in homoeostatic sleep pressure (Cajochen et al. 1999), differential performance on inhibitory tasks between chronotypes is homoeostatically controlled. Previous research has demonstrated that evening-types exhibit a slower build-up of sleep pressure and can tolerate a higher homoeostatic load compared to morning-types (Mongrain and Dumont 2007; Taillard et al. 2003). It is possible that this tolerance to homoeostatic sleep pressure enables evening-types to outperform morning-types on complex tasks such as resolving conflict. Further, morning-types have a faster increase in theta–alpha activity (an indicator of sleep pressure; ~6–9 Hz, Cajochen et al. 1995) during wakefulness compared to evening-types (Taillard et al. 2003). Increased theta activity, particularly in frontal brain regions, is associated with decreased attention (Klimesch 1999; Mann et al. 1992); and a larger proportion of alpha power in lower frequency bands has been reported in individuals with difficulty with inhibitory control (Crawford et al. 1995; Klimesch 1999). It is, therefore, possible that the elevation in theta–alpha activity across the waking day reduces attention, particularly inhibitory control, and that this effect is exaggerated in morning-types.

Contrastingly, it is possible that the preservation of executive control in those with a tendency towards eveningness relative to morningness is due to circadian phase differences between chronotypes. Circadian rhythms of core body temperature and secretion of melatonin are typically 2–3 h later in evening-types compared to morning-types (Lack et al. 2009). Previous studies have shown that daily rhythms of alertness often mimic the circadian profile of core body temperature (Akerstedt and Gillberg 1982; Baehr et al. 2000; Johnson et al. 1992; Kleitman and Jackson 1950; Monk et al. 1997). Enhanced performance often corresponds to higher core body temperature. During the evening, alertness and performance considerably decline, coinciding with the falling limb of the core body temperature rhythm (Dijk et al. 1992)—an effect which will inevitably occur later in evening-types. Thus, the preserved executive control in evening-types following 18 h of sustained wakefulness can be attributed to both changes in homoeostatic and circadian processes. Indeed, studies using constant routine or forced desynchrony paradigms have demonstrated the influence of both processes on variation in sleepiness and performance across the waking period (e.g. Dijk et al. 1992; Mollicone et al. 2008; Zhou et al. 2011). Further research from us focuses on disentangling the effects of the circadian rhythm and homoeostatic sleep drive on attentional functioning.

In sum, our results provide novel evidence that following 18 h of sustained wakefulness, the ability to resolve conflict is preserved in those with increasing tendency towards eveningness. However, it should be noted that this effect was evident only in the reaction time and error rate data, whereas the effect on the derived attentional network scores only bordered on significance. This finding contradicts that of Matchock and Mordkoff (2009) who found chronotype and time-of-day effects on the alerting network, and time-of-day effects on executive control. Certain limitations of the present study offer potential explanations for this discrepancy. First, our sample was not selected on the basis of extreme chronotype; rather, we examined the full chronotype spectrum. This was intentional as it allowed us to demonstrate that differential effects on attentional processing emerge even between less extreme variations in chronotype. As such, the present pattern may be conservative, and a replication of the current study including groups of extreme morning- and evening-types is likely to yield stronger effects. Second, this study was performed in participants’ homes with little control over potential confounding factors such as ambient lighting, temperature, posture, and food intake—all of which can in principle affect behavioural alertness and circadian rhythmicity (Duffy and Czeisler 2009; Kräuchi et al. 1997; Paz and Berry 1997; Waterhouse et al. 2005). Additionally, we did not take into account possible effects of “social jetlag” on attentional performance between chronotypes. It is possible that 8 am and 2 am test times do not reflect the same circadian phase between individuals, even for those with similar chronotypes, due to differential accumulation of sleep debt across the week prior to the sustained wakefulness protocol. During the working week, evening-types may incur greater sleep debt due to a greater mismatch between preferred sleep–wake times and those imposed by work or social obligations. This could account for the “asynchrony effect” observed in our RT data. Investigation of sleep–wake times obtained on work- versus free-days would provide a better representation of true circadian phase not confounded by social jetlag. Third relates to the fact that repeated testing using the ANT may induce practice effects. Ishigami and Klein (2010) demonstrated that practice effects are particularly potent in affecting executive function as assessed by the ANT. This is evidenced by decreased reaction time on incongruent trials with repeated testing. However, our data demonstrate *increased* RT at time 2 compared to time 1 (for 10/12 conditions). This pattern of results is inconsistent with a practice-effects explanation of our data, but rather demonstrates that the time effects observed are due to processes associated with sustained wakefulness. Furthermore, our time effects varied as a function of chronotype—a finding which cannot be explained by practice. That said, future studies would benefit from including a control group of participants who perform the ANT tasks in the reversed order (i.e. 2 am testing prior to 8 am testing) to confirm the absence of practice effects in the current context. Fourth, it is possible that our sample size was underpowered to detect significant interaction effects (particularly for the trends towards significance for the time × chronotype interactions on both orienting and conflict score). Thus, these results should be considered preliminary, and future studies should address these questions within larger samples. A final consideration is that, by measuring three components of attention within one test, the ANT may result in increased variation in response times due to the fact that each trial exhibits an alerting, orienting, and executive control component. Future research investigating the influence of chronotype and sustained wakefulness on attention would benefit from examining the sensitivity of pure measures of alerting, orienting, and executive control.

Overall, our results contribute to a better understanding of the effects of sleep deprivation, chronotype, and time-of-day effects on cognitive functioning, by considering these relationships across the time frame of a normal “socially constrained” waking day. We conclude that attentional processing is not holistically impaired following 18 h of sustained wakefulness, but rather specific attentional domains are impaired or preserved as a function of chronotype. These effects are likely driven by relative differences and changes in frontal functioning and dopaminergic activity between chronotypes across the waking day. These findings can be usefully translated to other areas of cognitive processing. For example, successful inhibition is necessary for numerous cognitive processes, including speech production and language comprehension, given that such processes require careful control over thought and action (Arbuckle and Gold 1993; Gernsbacher 1993; Logan and Cowan 1984). Thus, it is possible that these linguistic processes are modulated by chronotype and time-of-day in a similar manner as attention. On a wider scale, our results highlight that morning-types may be particularly vulnerable to failures in executive control in the later evening, which may have direct implications on risk of accidents particularly on tasks requiring inhibitory control. Future research from ourselves will investigate associations between biological indices of circadian rhythm phase, EEG signatures of increasing sleep pressure (i.e. theta–alpha activity; slow-wave activity), and attentional performance to provide insight into the components of attention that are affected by the circadian rhythm or homoeostatic mechanisms.
